# Supplementary material for: Hybrid assembly of an agricultural slurry virome reveals a diverse and stable community with the potential to alter the metabolism and virulence of veterinary pathogens
Source: Microbiome. 2021 Mar 20;9:65. doi: 10.1186/s40168-021-01010-3 (PMC7981956; doi:10.1186/s40168-021-01010-3)

Supplementary figure 1

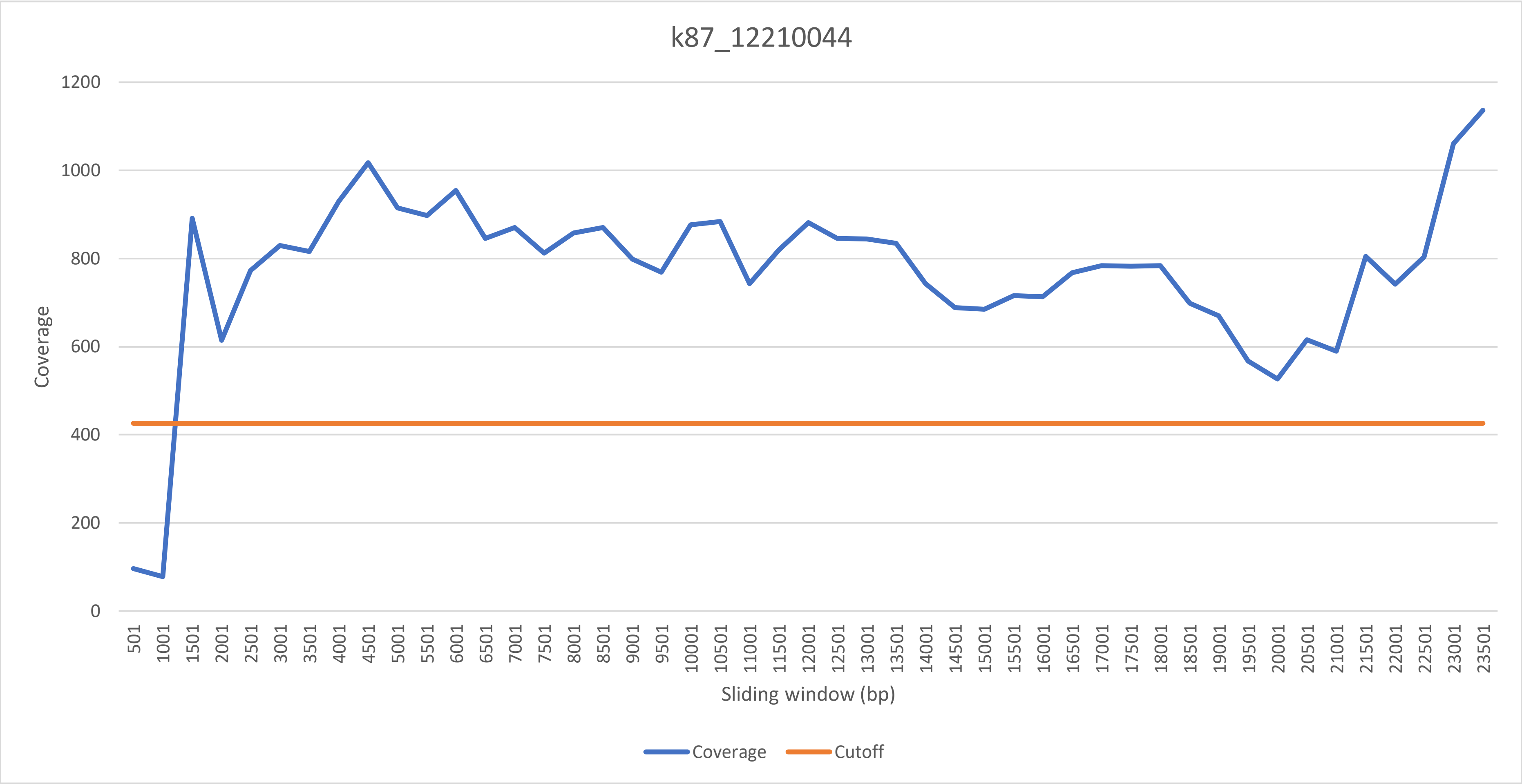

Supplementary figure 2

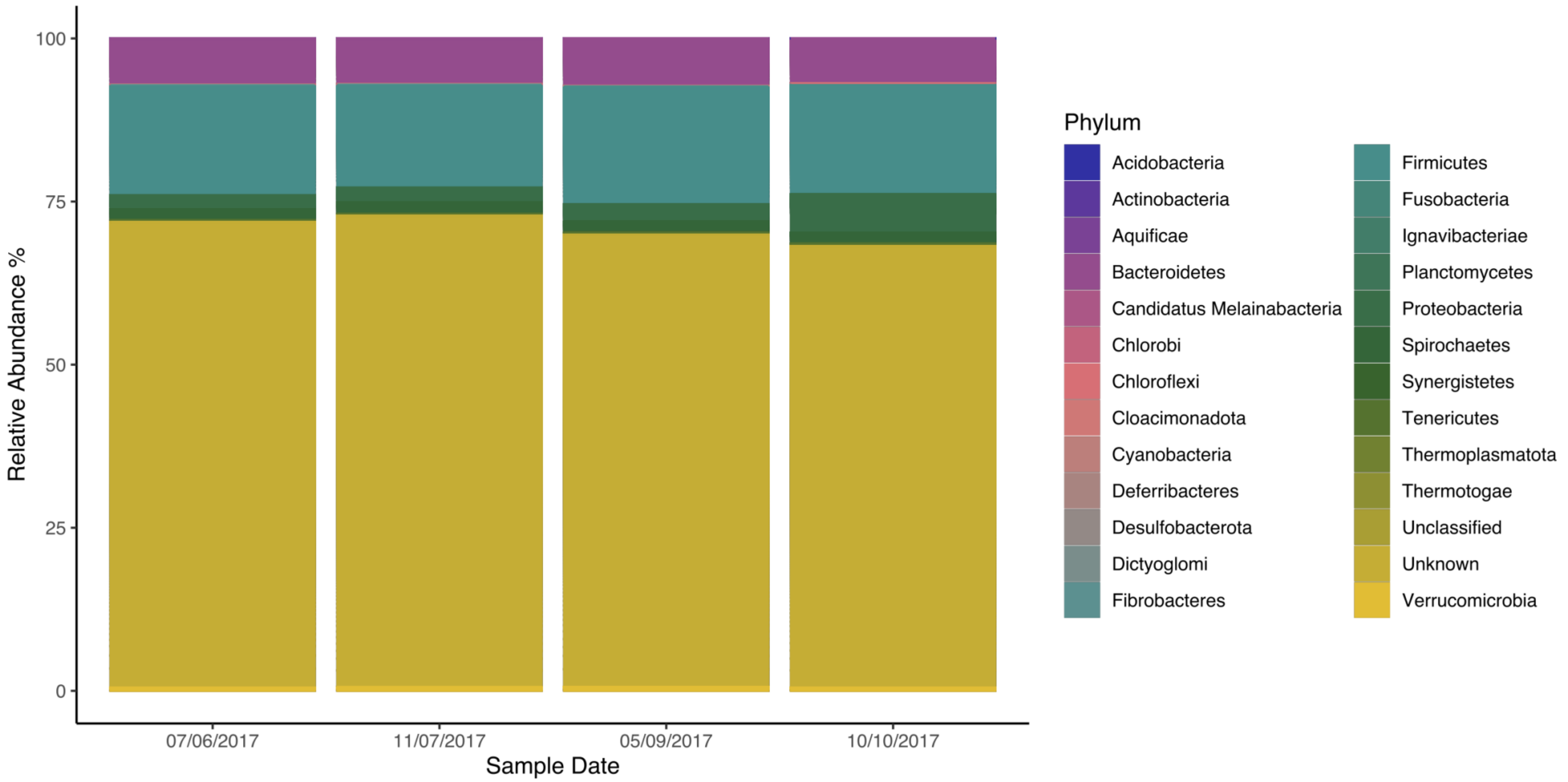

Supplementary figure 3

Primase

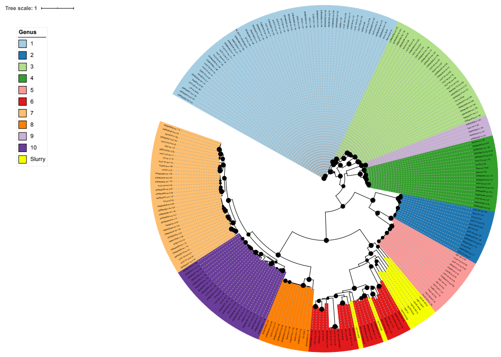

Terminase

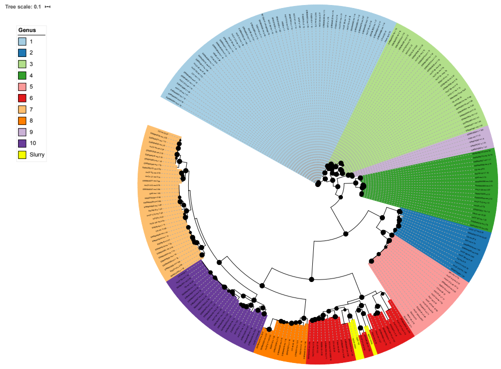

Portal

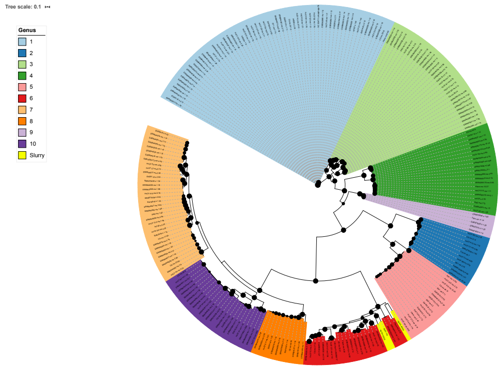

Major Capsid Protein

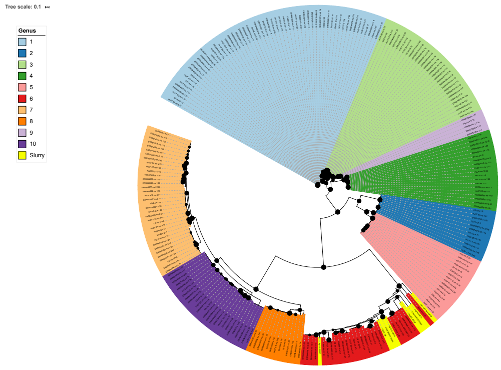

Supplementary figure 4

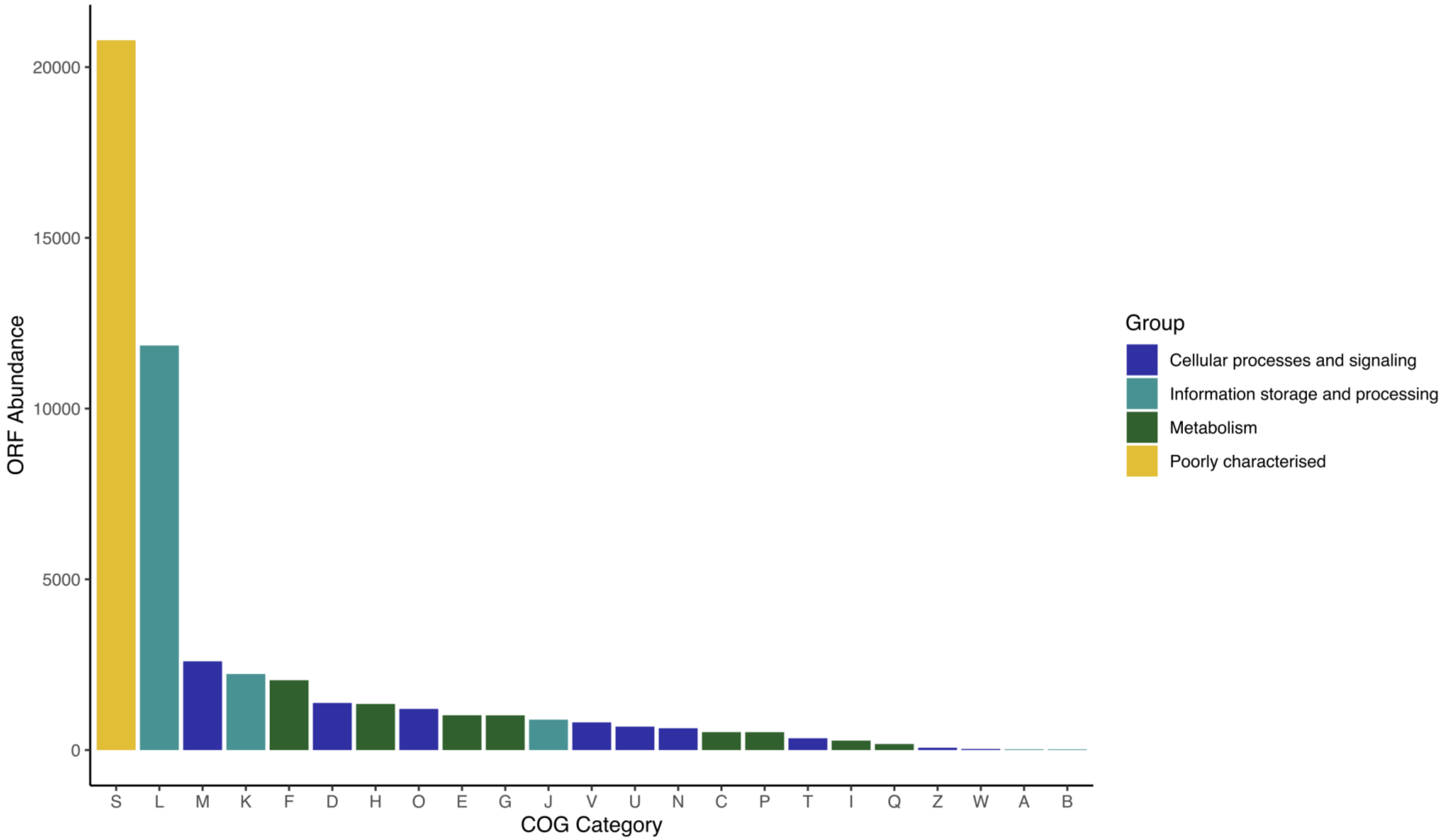

Supplementary figure 5

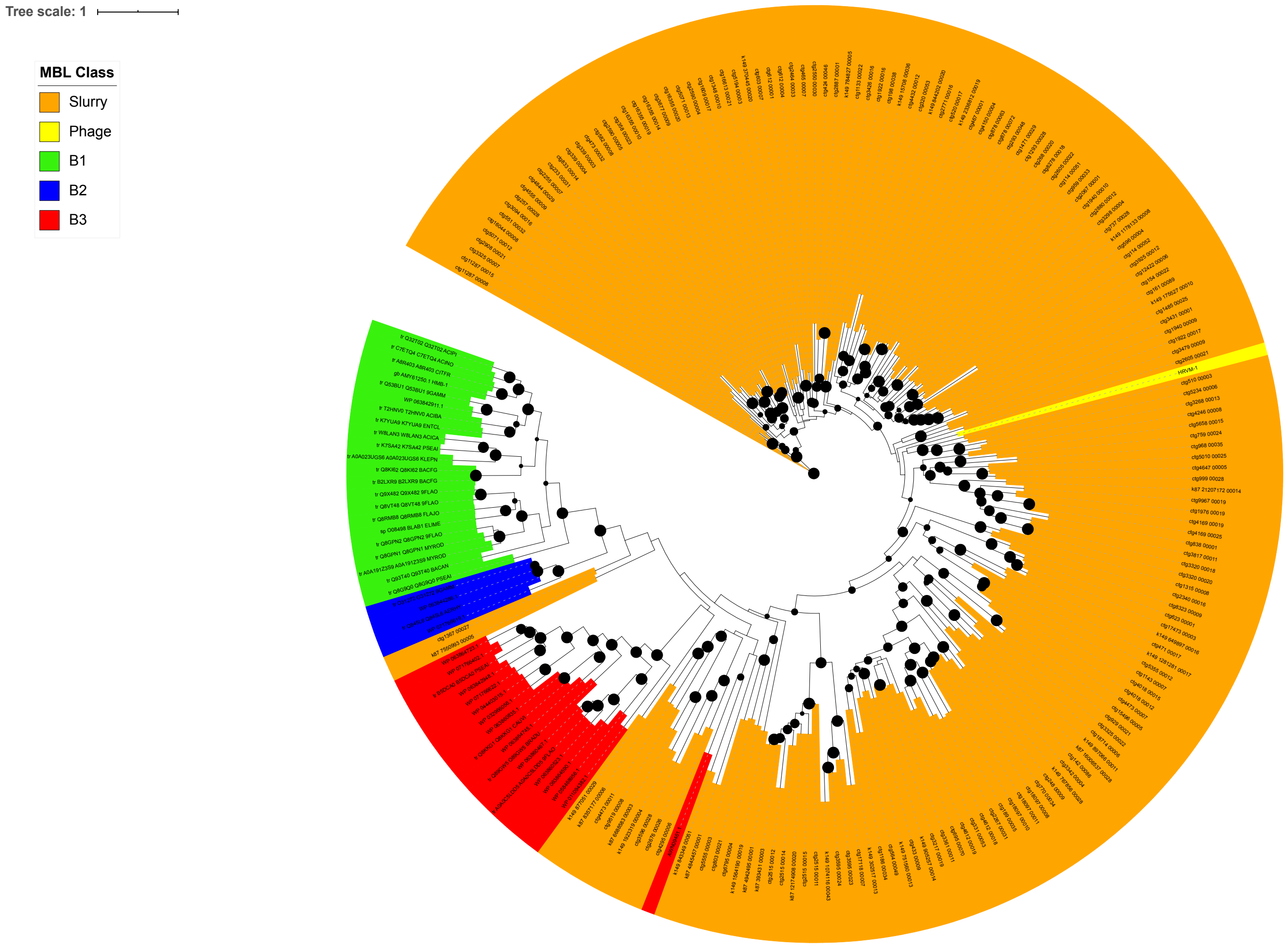

Supplementary figure 6

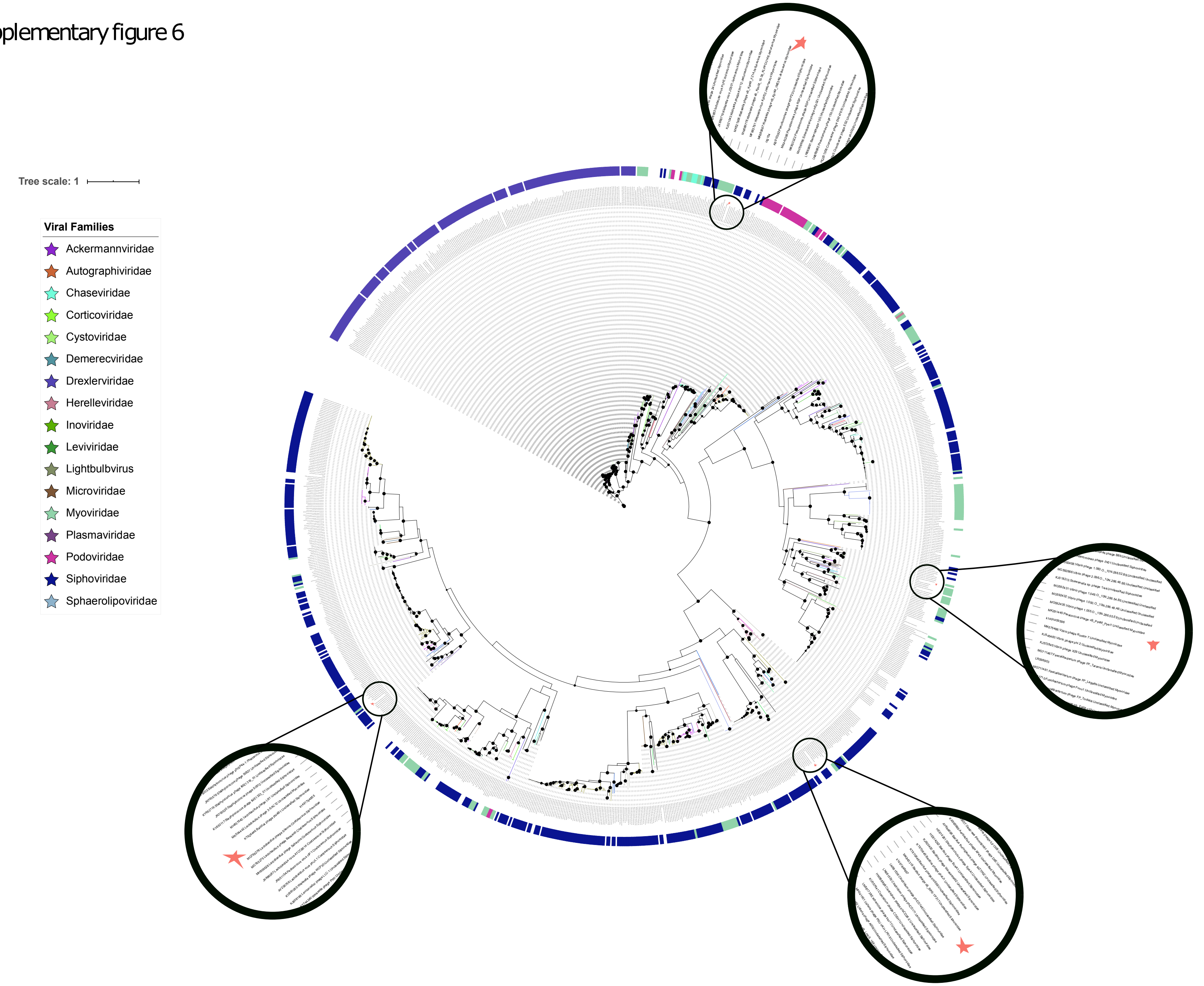

Supplement: Supplementary file 3 — Additional file 2: Supplementary Figure 1. Representative figure for the identification of prophage ends. Reads were mapped against vOTU k87_12210044 at 95 % identity threshold, the median coverage was calculated for 500 bp windows with the cutoff value calculated as median coverage minus (2 * standard deviations of median coverage) and plotted in orange. In this particular example, only one end was predicted. Supplementary Figure 2. Predicted hosts of viral contigs at the phylum level. Predicted hosts were obtained using WiSH. The relative abundance of phages predicted to infect different hosts was calculated by stringent mapping of reads to each viral contig as normalising for contig length and sequencing depth as described in materials and methods. Supplementary Figure 3. Phylogeny of crAss-like vOTUs based upon the method of Guerin et al. [47]. Phylogeny of four genes that encode a primase, terminase, portal protein and major capsid protein. The analysis followed the same method as described by Guerin et al. [47], with the ten major clades as previously defined marked. Bootstrap values >70% are marked by a circle. Supplementary Figure 4. Functional classification of viral proteins into COG categories by eggNOG mapping. Supplementary Figure 5. Phylogeny of putative metallo-β-lactamases. The phylogeny was built on the alignment of the amino acid sequences that were aligned by MAFFT. A WAG model of evolution was used in IQ-TREE with 1000 boostraps. Putative MBLs identified in the slurry tank are marked in orange, along with a previously experimentally validated phage-encoded MBL (yellow). Bacterial subclass B1 (green), B2 (blue), B3 (red) MBLs are also marked. Bootstrap values >70% are marked by a circle. Supplementary Figure 6. Phylogeny of phage genomes that contain a complete DGR. Phylogeny was constructed from the amino acid sequence of TerL protein that were aligned in mafft and phylogeny constructed with IQTREE with a WAG model of evolution and 1000 bootstrap [file 40168_2021_1010_MOESM3_ESM.pdf]
